# Supplementary material for: Machine learning on large scale perturbation screens for SARS-CoV-2 host factors identifies β-catenin/CBP inhibitor PRI-724 as a potent antiviral
Source: Front Microbiol. 2023 Jun 5;14:1193320. doi: 10.3389/fmicb.2023.1193320 (PMC10277617; doi:10.3389/fmicb.2023.1193320)
Supplement: Supplementary file 7 [file Data_Sheet_1.zip › SupplementaryMaterial/Kelch-et-al-Supplementary-Material_revised-RK.docx]

Supplementary Material

Machine learning on large scale perturbation screens for SARS-CoV-2 host factors identifies β-catenin/CBP inhibitor PRI-724 as a potent antiviral

**Maximilian A. Kelch^1^, Antonella Vera-Guapi^2^, Thomas Beder^3^, Marcus Oswald^4^, Alicia Hiemisch^4^, Nina Beil^5^, Piotr Wajda^5^, Sandra Ciesek^1,6,7^, Holger Erfle^5^, Tuna Toptan^1†^* and Rainer König^4†^***

^1^ Institute for Medical Virology, University Hospital Frankfurt, Goethe University Frankfurt, Frankfurt, Germany

^2^ Institute of Biochemistry II, University Hospital, Frankfurt am Main, Germany

^3^ Medical Department II, Hematology and Oncology, University Hospital Schleswig-Holstein, Kiel, Germany

^4^ Institute for Infectious Diseases and Infection Control, Jena University Hospital, Jena, Germany

^5^ Advanced Biological Screening Facility (ABSF), High-Content Analysis of the Cell (HiCell), BioQuant, Heidelberg University, Heidelberg, Germany

^6^ German Centre for Infection Research (DZIF), external partner site Frankfurt, Germany

^7^ Fraunhofer Institute for Translational Medicine and Pharmacology ITMP, Frankfurt am Main, Germany

^†^ These authors have contributed equally to this work and share last authorship

*** Correspondence:**Tuna Toptan
tuna.toptangrabmair@kgu.de

Rainer König

rainer.koenig@uni-jena.de

**
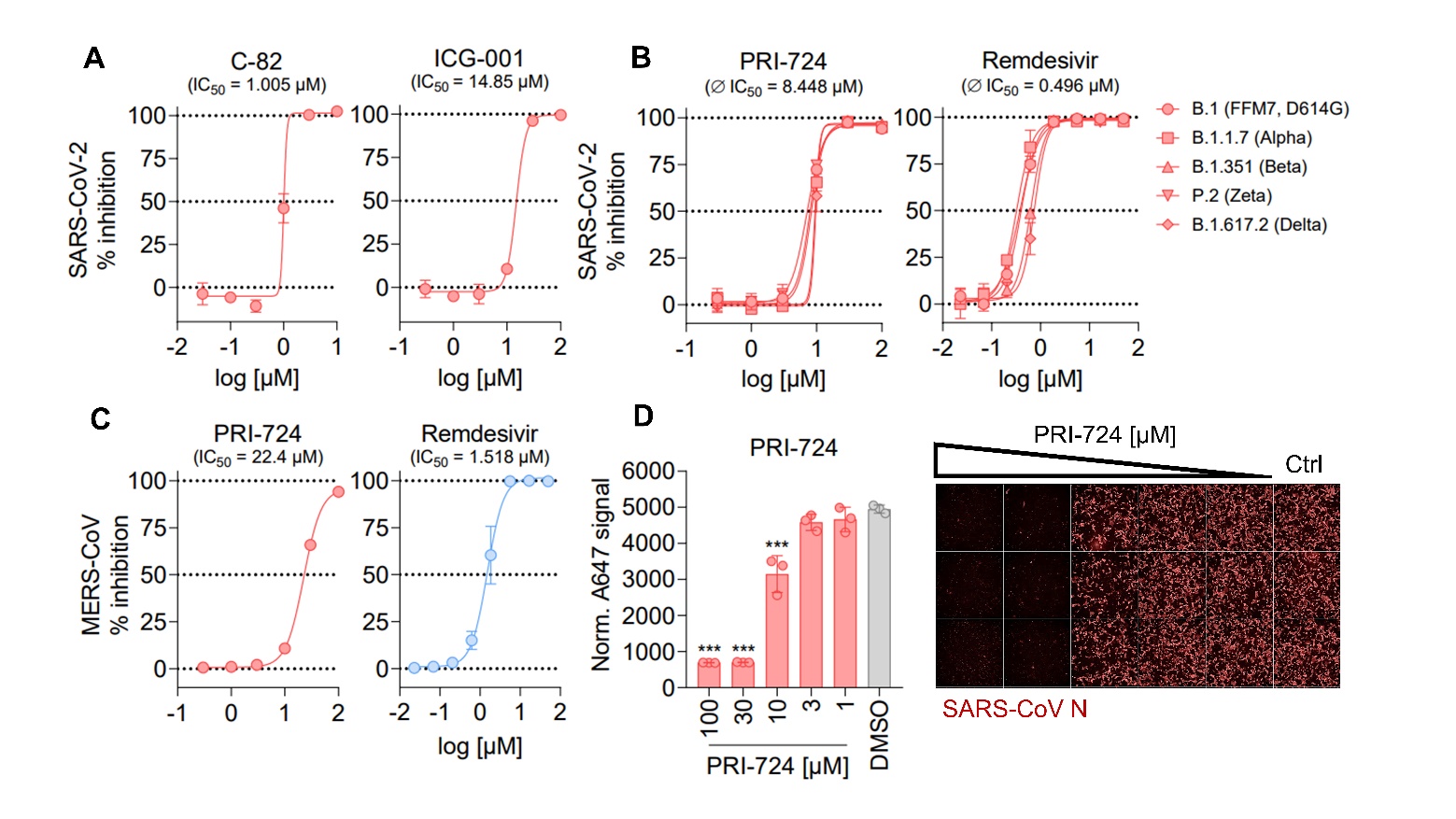
**

**Supplementary Figure S1:** Effects of PRI-724-related drugs C-82 and ICG-001 and PRI-724 effects on SARS-CoV-2/-1 and MERS-CoV in Calu-3 **(A)** A549-AT cells were treated with escalating doses of C-82 (left) and ICG-001 (right) and were infected with SARS-CoV-2 B.1.617.2 (MOI=0.1) for 48 h. Confluence of cells was determined using Spark Cyto**.** Data represent mean and SD of three biological replicates. The experiment was repeated twice showing similar results. **(B)** Calu-3 cells were treated with escalating doses of PRI-724 and Remdesivir and were subsequently infected with SARS-CoV-2 variants B.1 (circle), B.1.1.7 (square), B.1.351 (triangle up), P.2 (triangle down) and B.1.617.2 (rhombus) (MOI=0.1) for 72 h. Confluence of cells was determined using Spark Cyto**.** Data represent mean and SD of three biological replicates. The experiment was repeated twice showing similar results. **(C)** Calu-3 cells were treated with escalating doses of PRI-724 and Remdesivir and were infected with MERS-CoV (MOI=0.1) for 72 h. Confluence of cells was determined using Spark Cyto**.** Data represent mean and SD of three biological replicates. The experiment was repeated twice showing similar results. **(D)** Calu-3 cells were treated with escalating doses of PRI-724 and were infected with SARS-CoV-1 (MOI=0.1) for 72 h. Cells were incubated with antibody PA1 and were stained with antibody SA1 (**Supplementary Table S11**). Data represent mean and SD of three biological replicates. The experiment was repeated twice showing similar results.


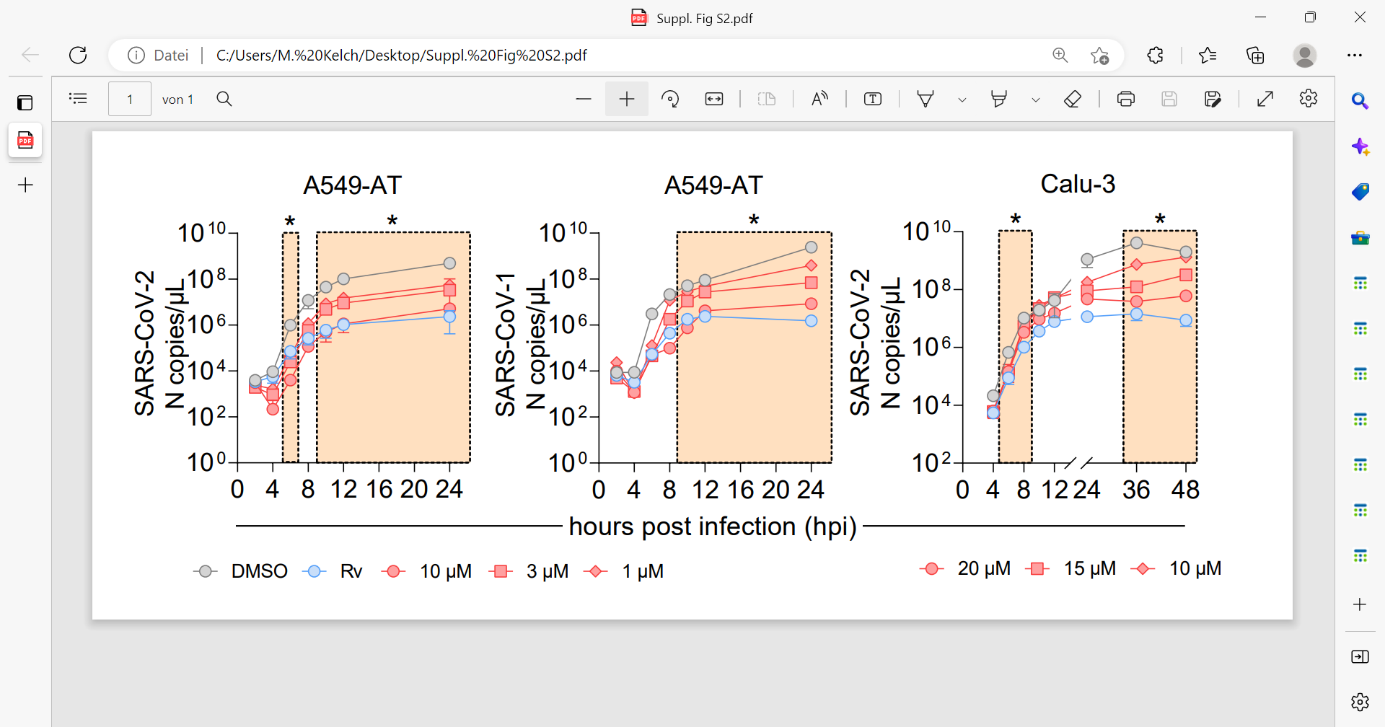


**Supplementary Figure S2:** N gene RNA kinetics of SARS-CoV-2 and SARS-CoV-1 in A549-AT and of SARS-CoV-2 in Calu-3**.** Cells were treated with different doses of PRI-724, 3 µM Remdesivir and DMSO and were subsequently infected with SARS-CoV-2 B.1.617.2 and SARS-CoV-1 (FFM1) (MOI=0.1) for 24 h. Calu-3 cells were treated for 48 h. RNA was isolated at indicated time points and N RNA was measured by qRT-PCR using primers P4, P5, P6, P10, P11 and P12. Data represent mean and SD of three biological replicates. The experiment was repeated twice showing similar results. Significant results of two-way ANOVA are indicated by asterisks; *p*<0.05.


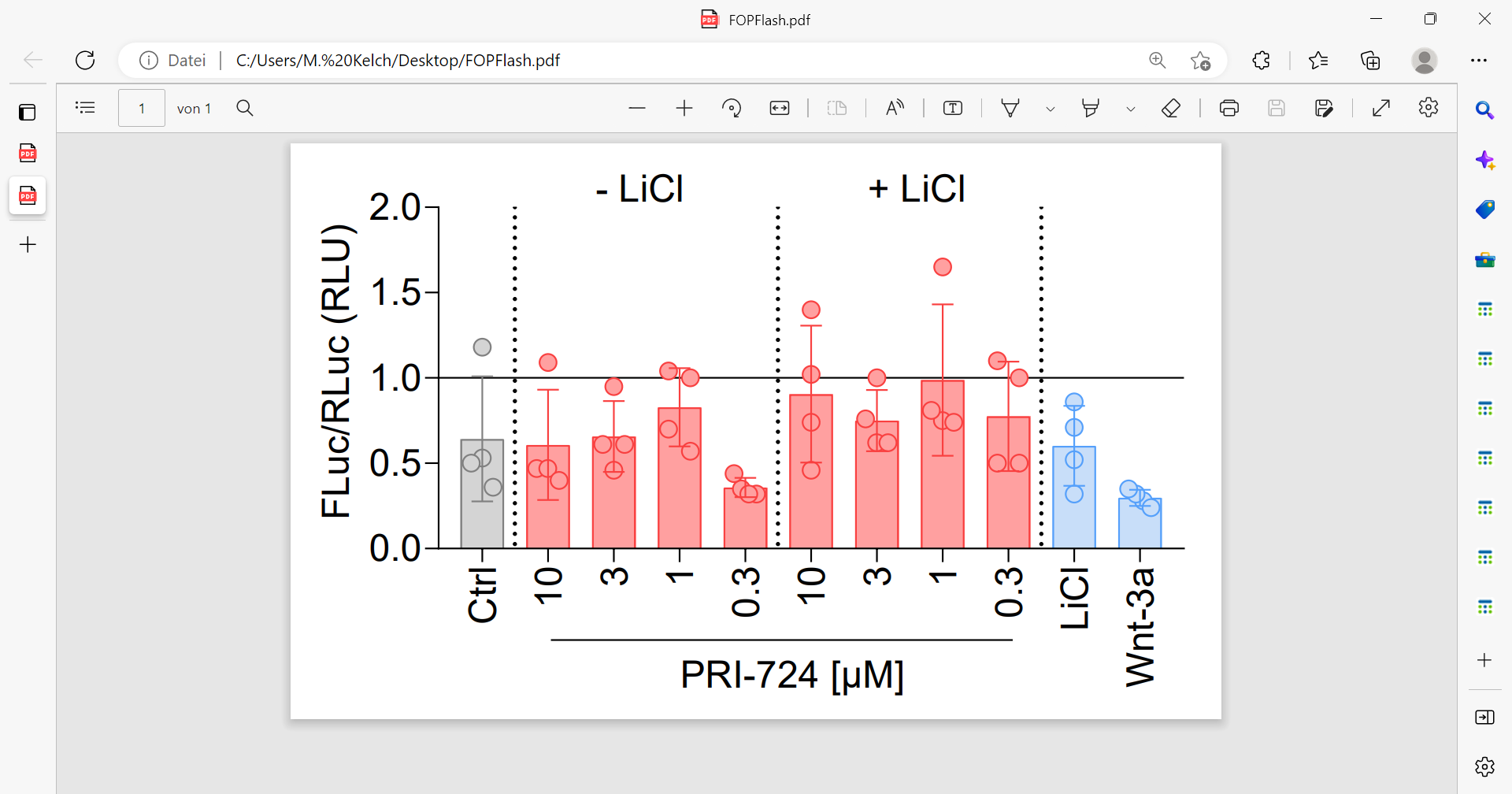


**Supplementary Figure S3:** FOPFlash reporter activity upon treatment with PRI-724 and Wnt-3a/LiCl**.** A549-AT cells were transfected with M51 Super 8x FOPFlash and pRL-SV40 and thereafter treated with 10 µM, 3 µM, 1 µM and 0.3 µM PRI-724 (red), 10 mM LiCl, rhWnt-3a (blue) or DMSO (grey). Luciferase activity was measured 24 h post treatment using Dual Luciferase Reporter Assay Kit (Promega) according to manufacturer’s instructions. Data represent mean and SD of four biological replicates.

Supplementary Figure S4: Cell count for indicated PRI-724 concentrations in A549-AT cells obtained by staining with Hoechst 33342. Cells were fixed and stained 48 h post treatment. Cells NC:Cells in pure medium; DMSO NC: Cells with the highest DMSO amount, as control for the treatment with PRI-724.


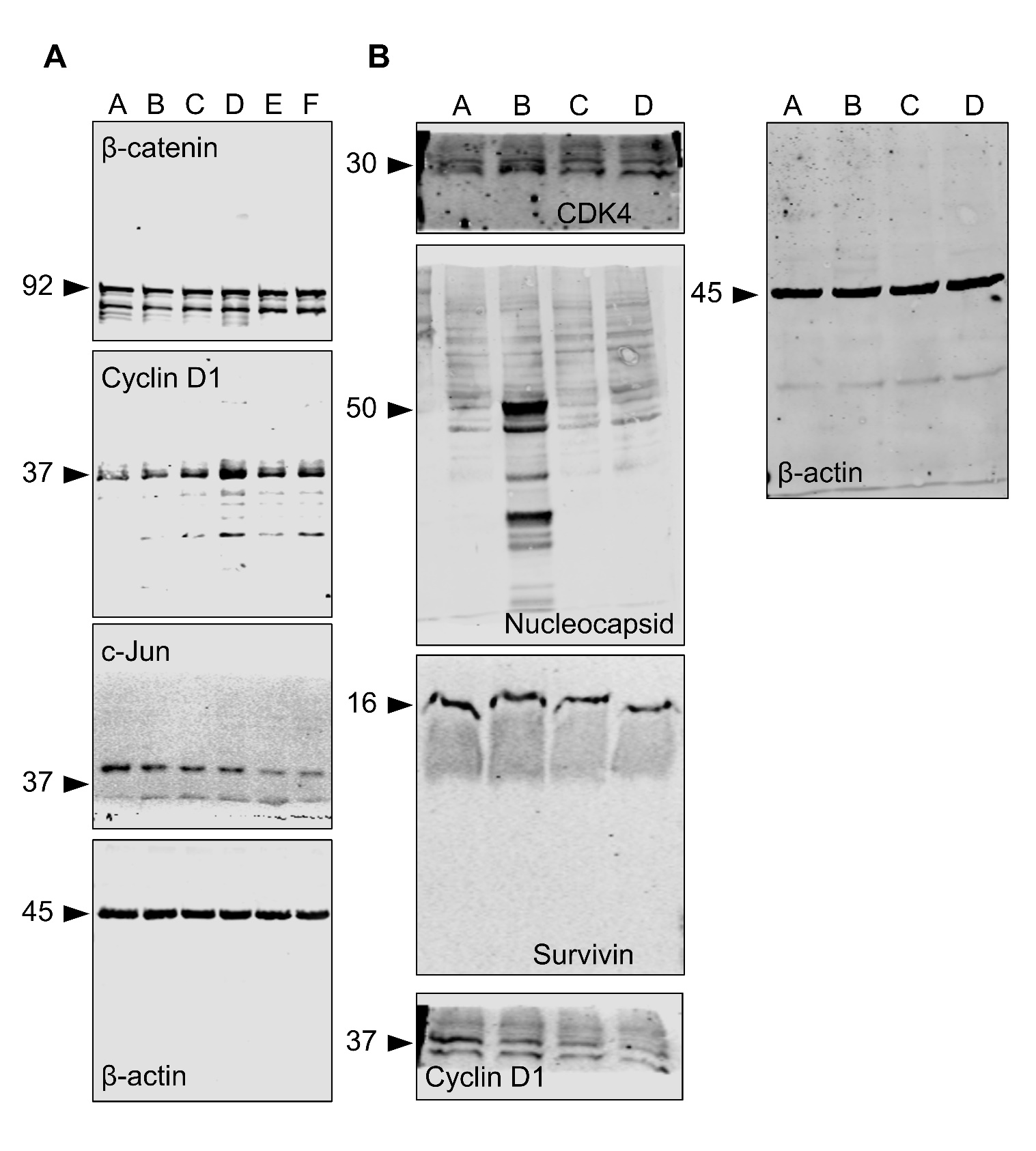


**Supplementary Figure S5:** Full-length western blots. **(A)** Western blots presented in Figure 6C. Numbers indicate molecular weight in kDa. Lane A – 10 µM PRI-724, Lane B – 3 µM PRI-724, Lane C – 1 µM PRI-724, Lane D – 0.3 µM PRI-724, Lane E – 3 µM Remdesivir, Lane F – DMSO. (B) Western blots presented in Figure 7B. Numbers indicate molecular weight in kDa. Lane A – DMSO/Mock, Lane B – DMSO/SARS-CoV-2 MOI 0.01, Lane C – 10 µM PRI-724/Mock, Lane D – 10 µM PRI-724/SARS-CoV-2 MOI 0.01. For experimental information refer to Material and methods as well as Figure 6C and 7B.

**Supplementary Table S1:** Overlapping gene sets among the investigated knockout screens

| GO.ID | Term Name | Daniloski | Wei | Wang | Zhu | Intersection |
| --- | --- | --- | --- | --- | --- | --- |
| GO:0090382 | phagosome maturation | 1 | 1 | 0 | 1 | 3 |
| GO:0000041 | transition metal ion transport | 1 | 0 | 1 | 1 | 3 |
| GO:0098876 | vesicle-mediated transport to the plasma membrane | 1 | 0 | 1 | 1 | 3 |
| GO:0007040 | lysosome organization | 1 | 0 | 1 | 1 | 3 |
| GO:0080171 | lytic vacuole organization | 1 | 0 | 1 | 1 | 3 |
| GO:0008333 | endosome to lysosome transport | 1 | 1 | 0 | 1 | 3 |
| GO:0071604 | transforming growth factor beta production | 1 | 1 | 1 | 0 | 3 |
| GO:0006892 | post-Golgi vesicle-mediated transport | 1 | 0 | 1 | 1 | 3 |
| GO:0007369 | gastrulation | 0 | 1 | 1 | 1 | 3 |
| GO:0030308 | negative regulation of cell growth | 0 | 1 | 1 | 1 | 3 |
| GO:0046488 | phosphatidylinositol metabolic process | 0 | 1 | 1 | 1 | 3 |
| GO:0006661 | phosphatidylinositol biosynthetic process | 0 | 1 | 1 | 1 | 3 |
| GO:0016469 | proton-transporting two-sector ATPase complex | 1 | 0 | 0 | 1 | 2 |
| GO:0033176 | proton-transporting V-type ATPase complex | 1 | 0 | 0 | 1 | 2 |
| GO:0090383 | phagosome acidification | 1 | 0 | 0 | 1 | 2 |
| GO:0009678 | pyrophosphate hydrolysis-driven proton transmembrane transporter activity | 1 | 0 | 0 | 1 | 2 |
| GO:0033572 | transferrin transport | 1 | 0 | 0 | 1 | 2 |
| GO:0045851 | pH reduction | 1 | 0 | 0 | 1 | 2 |
| GO:0044769 | ATPase activity coupled to transmembrane movement of ions rotational mechanism | 1 | 0 | 0 | 1 | 2 |
| GO:0046961 | proton-transporting ATPase activity rotational mechanism | 1 | 0 | 0 | 1 | 2 |
| GO:0051452 | intracellular pH reduction | 1 | 0 | 0 | 1 | 2 |
| GO:0006826 | iron ion transport | 1 | 0 | 0 | 1 | 2 |
| GO:0006885 | regulation of pH | 1 | 0 | 0 | 1 | 2 |
| GO:0030641 | regulation of cellular pH | 1 | 0 | 0 | 1 | 2 |
| GO:0055067 | monovalent inorganic cation homeostasis | 1 | 0 | 0 | 1 | 2 |
| GO:0038094 | Fc-gamma receptor signaling pathway | 1 | 0 | 0 | 1 | 2 |
| GO:0016471 | vacuolar proton-transporting V-type ATPase complex | 1 | 0 | 0 | 1 | 2 |
| GO:0033178 | proton-transporting two-sector ATPase complex catalytic domain | 1 | 0 | 0 | 1 | 2 |
| GO:0002433 | immune response-regulating cell surface receptor signaling pathway involved in phagocytosis | 1 | 0 | 0 | 1 | 2 |
| GO:0038096 | Fc-gamma receptor signaling pathway involved in phagocytosis | 1 | 0 | 0 | 1 | 2 |
| GO:0005885 | Arp2/3 protein complex | 1 | 0 | 0 | 1 | 2 |
| GO:0016241 | regulation of macroautophagy | 1 | 0 | 0 | 1 | 2 |
| GO:0019829 | ATPase-coupled cation transmembrane transporter activity | 1 | 0 | 0 | 1 | 2 |
| GO:0002431 | Fc receptor mediated stimulatory signaling pathway | 1 | 0 | 0 | 1 | 2 |
| GO:0030904 | retromer complex | 1 | 0 | 0 | 1 | 2 |
| GO:0015078 | proton transmembrane transporter activity | 1 | 0 | 0 | 1 | 2 |
| GO:0036295 | cellular response to increased oxygen levels | 1 | 0 | 0 | 1 | 2 |
| GO:0042625 | ATPase-coupled ion transmembrane transporter activity | 1 | 0 | 0 | 1 | 2 |
| GO:0034314 | Arp2/3 complex-mediated actin nucleation | 1 | 0 | 0 | 1 | 2 |
| GO:0046916 | cellular transition metal ion homeostasis | 1 | 0 | 0 | 1 | 2 |
| GO:0016482 | cytosolic transport | 1 | 0 | 0 | 1 | 2 |
| GO:1990126 | retrograde transport, endosome to plasma membrane | 1 | 0 | 0 | 1 | 2 |
| GO:0007034 | vacuolar transport | 1 | 0 | 0 | 1 | 2 |
| GO:0008064 | regulation of actin polymerization or depolymerization | 1 | 0 | 0 | 1 | 2 |
| GO:0030832 | regulation of actin filament length | 1 | 0 | 0 | 1 | 2 |
| GO:0008286 | insulin receptor signaling pathway | 1 | 0 | 0 | 1 | 2 |
| GO:1901099 | negative regulation of signal transduction in absence of ligand | 1 | 0 | 0 | 1 | 2 |
| GO:2001240 | negative regulation of extrinsic apoptotic signaling pathway in absence of ligand | 1 | 0 | 0 | 1 | 2 |
| GO:0007041 | lysosomal transport | 1 | 0 | 0 | 1 | 2 |
| GO:0048013 | ephrin receptor signaling pathway | 1 | 0 | 0 | 1 | 2 |
| GO:0050879 | multicellular organismal movement | 1 | 0 | 0 | 1 | 2 |
| GO:0050881 | musculoskeletal movement | 1 | 0 | 0 | 1 | 2 |
| GO:2001239 | regulation of extrinsic apoptotic signaling pathway in absence of ligand | 1 | 0 | 0 | 1 | 2 |
| GO:0005200 | structural constituent of cytoskeleton | 1 | 0 | 0 | 1 | 2 |
| GO:0048813 | dendrite morphogenesis | 1 | 0 | 0 | 1 | 2 |
| GO:1901021 | positive regulation of calcium ion transmembrane transporter activity | 1 | 0 | 0 | 1 | 2 |
| GO:0015012 | heparan sulfate proteoglycan biosynthetic process | 0 | 0 | 1 | 1 | 2 |
| GO:0031526 | brush border membrane | 0 | 0 | 1 | 1 | 2 |
| GO:0008157 | protein phosphatase 1 binding | 0 | 0 | 1 | 1 | 2 |
| GO:0008287 | protein serine/threonine phosphatase complex | 0 | 0 | 1 | 1 | 2 |
| GO:1903293 | phosphatase complex | 0 | 0 | 1 | 1 | 2 |
| GO:0007519 | skeletal muscle tissue development | 0 | 0 | 1 | 1 | 2 |
| GO:0042552 | myelination | 0 | 0 | 1 | 1 | 2 |
| GO:0006023 | aminoglycan biosynthetic process | 0 | 0 | 1 | 1 | 2 |
| GO:0007272 | ensheathment of neurons | 0 | 0 | 1 | 1 | 2 |
| GO:0008366 | axon ensheathment | 0 | 0 | 1 | 1 | 2 |
| GO:0098685 | Schaffer collateral - CA1 synapse | 0 | 0 | 1 | 1 | 2 |
| GO:0005903 | brush border | 0 | 0 | 1 | 1 | 2 |
| GO:0070262 | peptidyl-serine dephosphorylation | 0 | 0 | 1 | 1 | 2 |
| GO:0019888 | protein phosphatase regulator activity | 0 | 0 | 1 | 1 | 2 |
| GO:0045667 | regulation of osteoblast differentiation | 0 | 0 | 1 | 1 | 2 |
| GO:0045722 | positive regulation of gluconeogenesis | 0 | 0 | 1 | 1 | 2 |
| GO:0045995 | regulation of embryonic development | 0 | 0 | 1 | 1 | 2 |
| GO:0001968 | fibronectin binding | 0 | 0 | 1 | 1 | 2 |
| GO:0006878 | cellular copper ion homeostasis | 0 | 0 | 1 | 1 | 2 |
| GO:0032007 | negative regulation of TOR signaling | 0 | 0 | 1 | 1 | 2 |
| GO:0071542 | dopaminergic neuron differentiation | 0 | 0 | 1 | 1 | 2 |
| GO:0006024 | glycosaminoglycan biosynthetic process | 0 | 0 | 1 | 1 | 2 |
| GO:0008207 | C21-steroid hormone metabolic process | 0 | 0 | 1 | 1 | 2 |
| GO:0019208 | phosphatase regulator activity | 0 | 0 | 1 | 1 | 2 |
| GO:0098862 | cluster of actin-based cell projections | 0 | 0 | 1 | 1 | 2 |
| GO:0004722 | protein serine/threonine phosphatase activity | 0 | 0 | 1 | 1 | 2 |
| GO:0055070 | copper ion homeostasis | 0 | 0 | 1 | 1 | 2 |
| GO:0048284 | organelle fusion | 1 | 1 | 0 | 0 | 2 |
| GO:0097352 | autophagosome maturation | 1 | 1 | 0 | 0 | 2 |
| GO:0000421 | autophagosome membrane | 1 | 1 | 0 | 0 | 2 |
| GO:0006906 | vesicle fusion | 1 | 1 | 0 | 0 | 2 |
| GO:0042983 | amyloid precursor protein biosynthetic process | 1 | 1 | 0 | 0 | 2 |
| GO:0042984 | regulation of amyloid precursor protein biosynthetic process | 1 | 1 | 0 | 0 | 2 |
| GO:0090174 | organelle membrane fusion | 1 | 1 | 0 | 0 | 2 |
| GO:0015605 | organophosphate ester transmembrane transporter activity | 1 | 1 | 0 | 0 | 2 |
| GO:0007033 | vacuole organization | 1 | 1 | 0 | 0 | 2 |
| GO:0071634 | regulation of transforming growth factor beta production | 1 | 1 | 0 | 0 | 2 |
| GO:0010812 | negative regulation of cell-substrate adhesion | 1 | 1 | 0 | 0 | 2 |
| GO:0061025 | membrane fusion | 1 | 1 | 0 | 0 | 2 |
| GO:0110020 | regulation of actomyosin structure organization | 1 | 1 | 0 | 0 | 2 |
| GO:0035493 | SNARE complex assembly | 1 | 1 | 0 | 0 | 2 |
| GO:0006941 | striated muscle contraction | 1 | 1 | 0 | 0 | 2 |
| GO:0043550 | regulation of lipid kinase activity | 1 | 1 | 0 | 0 | 2 |
| GO:0030897 | HOPS complex | 1 | 1 | 0 | 0 | 2 |
| GO:0086005 | ventricular cardiac muscle cell action potential | 1 | 1 | 0 | 0 | 2 |
| GO:0001952 | regulation of cell-matrix adhesion | 1 | 1 | 0 | 0 | 2 |
| GO:0010665 | regulation of cardiac muscle cell apoptotic process | 1 | 1 | 0 | 0 | 2 |
| GO:0030136 | clathrin-coated vesicle | 1 | 1 | 0 | 0 | 2 |
| GO:0006862 | nucleotide transport | 1 | 1 | 0 | 0 | 2 |
| GO:0001953 | negative regulation of cell-matrix adhesion | 1 | 1 | 0 | 0 | 2 |
| GO:0015216 | purine nucleotide transmembrane transporter activity | 1 | 1 | 0 | 0 | 2 |
| GO:0051492 | regulation of stress fiber assembly | 1 | 1 | 0 | 0 | 2 |
| GO:0060048 | cardiac muscle contraction | 1 | 1 | 0 | 0 | 2 |
| GO:1901998 | toxin transport | 1 | 1 | 0 | 0 | 2 |
| GO:0005942 | phosphatidylinositol 3-kinase complex | 1 | 0 | 1 | 0 | 2 |
| GO:0006904 | vesicle docking involved in exocytosis | 1 | 0 | 1 | 0 | 2 |
| GO:0007260 | tyrosine phosphorylation of STAT protein | 1 | 0 | 1 | 0 | 2 |
| GO:0022617 | extracellular matrix disassembly | 1 | 0 | 1 | 0 | 2 |
| GO:0030027 | lamellipodium | 1 | 0 | 1 | 0 | 2 |
| GO:0034614 | cellular response to reactive oxygen species | 1 | 0 | 1 | 0 | 2 |
| GO:0048278 | vesicle docking | 1 | 0 | 1 | 0 | 2 |
| GO:0042542 | response to hydrogen peroxide | 1 | 0 | 1 | 0 | 2 |
| GO:0048538 | thymus development | 1 | 0 | 1 | 0 | 2 |
| GO:0140029 | exocytic process | 1 | 0 | 1 | 0 | 2 |
| GO:0003044 | regulation of systemic arterial blood pressure mediated by a chemical signal | 1 | 0 | 1 | 0 | 2 |
| GO:0001990 | regulation of systemic arterial blood pressure by hormone | 1 | 0 | 1 | 0 | 2 |
| GO:0001764 | neuron migration | 1 | 0 | 1 | 0 | 2 |
| GO:0050796 | regulation of insulin secretion | 1 | 0 | 1 | 0 | 2 |
| GO:0044344 | cellular response to fibroblast growth factor stimulus | 1 | 0 | 1 | 0 | 2 |
| GO:0098686 | hippocampal mossy fiber to CA3 synapse | 1 | 0 | 1 | 0 | 2 |
| GO:0048641 | regulation of skeletal muscle tissue development | 1 | 0 | 1 | 0 | 2 |
| GO:0016248 | channel inhibitor activity | 1 | 0 | 1 | 0 | 2 |
| GO:0050886 | endocrine process | 1 | 0 | 1 | 0 | 2 |
| GO:0006491 | N-glycan processing | 1 | 0 | 1 | 0 | 2 |
| GO:0071774 | response to fibroblast growth factor | 1 | 0 | 1 | 0 | 2 |
| GO:0035257 | nuclear hormone receptor binding | 0 | 1 | 1 | 0 | 2 |
| GO:0051427 | hormone receptor binding | 0 | 1 | 1 | 0 | 2 |
| GO:0001704 | formation of primary germ layer | 0 | 1 | 1 | 0 | 2 |
| GO:0034644 | cellular response to UV | 0 | 1 | 1 | 0 | 2 |
| GO:0010224 | response to UV-B | 0 | 1 | 1 | 0 | 2 |
| GO:0007492 | endoderm development | 0 | 1 | 1 | 0 | 2 |
| GO:0033522 | histone H2A ubiquitination | 0 | 1 | 1 | 0 | 2 |
| GO:0000578 | embryonic axis specification | 0 | 1 | 1 | 0 | 2 |
| GO:0035004 | phosphatidylinositol 3-kinase activity | 0 | 1 | 1 | 0 | 2 |
| GO:0030414 | peptidase inhibitor activity | 0 | 1 | 1 | 0 | 2 |
| GO:0036092 | phosphatidylinositol-3-phosphate biosynthetic process | 0 | 1 | 1 | 0 | 2 |
| GO:0014744 | positive regulation of muscle adaptation | 0 | 1 | 1 | 0 | 2 |
| GO:0071236 | cellular response to antibiotic | 0 | 1 | 1 | 0 | 2 |
| GO:0016307 | phosphatidylinositol phosphate kinase activity | 0 | 1 | 1 | 0 | 2 |
| GO:0030218 | erythrocyte differentiation | 0 | 1 | 1 | 0 | 2 |
| GO:0004866 | endopeptidase inhibitor activity | 0 | 1 | 1 | 0 | 2 |

**Supplementary Table S2: Performance measures of the two classifiers**

|  | Mean knockout screens based classifier | Standard deviation knockout screens based classifier | Mean drug screen based classifier | Standard deviation drug screen based classifier |
| --- | --- | --- | --- | --- |
| Sensitivity/Recall | 0.71 | 0.12 | 0.32 | 0.12 |
| Specificity | 0.76 | 0.06 | 0.93 | 0.01 |
| Precision | 0.62 | 0.03 | 0.31 | 0.08 |
| F1 | 0.66 | 0.06 | 0.32 | 0.10 |
| Balanced Accuracy | 0.73 | 0.04 | 0.62 | 0.06 |
| ROCAUC | 0.82 | 0.03 | 0.71 | 0.11 |

**Supplementary Tables 3 and 4 are provided in separate files**

**Supplementary Table S5:** Gene sets with enriched predicted HDF of the knockout screens *and* the drugs screen based classifiers^*^

| **GO ID** | **Term name** |
| --- | --- |
| GO:0007369 | gastrulation |
| GO:0001653 | peptide receptor activity |
| GO:0007200 | phospholipase C-activating G protein-coupled receptor signaling pathway |
| GO:0030594 | neurotransmitter receptor activity |
| GO:0019955 | cytokine binding |
| GO:0050731 | positive regulation of peptidyl-tyrosine phosphorylation |
| GO:0008217 | regulation of blood pressure |
| GO:0007193 | adenylate cyclase-inhibiting G protein-coupled receptor signaling pathway |
| GO:0003018 | vascular process in circulatory system |
| GO:0006939 | smooth muscle contraction |
| GO:0019933 | cAMP-mediated signaling |
| GO:0048771 | tissue remodeling |
| GO:0050921 | positive regulation of chemotaxis |
| GO:0051897 | positive regulation of protein kinase B signaling |
| GO:0098982 | GABA-ergic synapse |
| GO:0070851 | growth factor receptor binding |
| GO:0048167 | regulation of synaptic plasticity |
| GO:0019838 | growth factor binding |
| GO:0015085 | calcium ion transmembrane transporter activity |
| GO:0008066 | glutamate receptor activity |
| GO:0015079 | potassium ion transmembrane transporter activity |
| GO:0019233 | sensory perception of pain |
| GO:0045778 | positive regulation of ossification |
| GO:0046717 | acid secretion |
| GO:0048017 | inositol lipid-mediated signaling |
| GO:0042698 | ovulation cycle |
| GO:0001508 | action potential |
| GO:0051932 | synaptic transmission, GABAergic |
| GO:0007613 | memory |
| GO:0007612 | learning |
| GO:0008277 | regulation of G protein-coupled receptor signaling pathway |
| GO:0010518 | positive regulation of phospholipase activity |
| GO:0007416 | synapse assembly |
| GO:0031644 | regulation of nervous system process |
| GO:0030534 | adult behavior |
| GO:0042165 | neurotransmitter binding |
| GO:0019226 | transmission of nerve impulse |
| GO:0043197 | dendritic spine |
| GO:0098739 | import across plasma membrane |
| GO:0022617 | extracellular matrix disassembly |
| GO:0050796 | regulation of insulin secretion |
| GO:0043204 | perikaryon |
| GO:0000187 | activation of MAPK activity |
| GO:0050795 | regulation of behavior |
| GO:0048660 | regulation of smooth muscle cell proliferation |
| GO:0061138 | morphogenesis of a branching epithelium |
| GO:0099528 | G protein-coupled neurotransmitter receptor activity |
| GO:0007631 | feeding behavior |
| GO:2001257 | regulation of cation channel activity |
| GO:0014074 | response to purine-containing compound |
| GO:0001659 | temperature homeostasis |
| GO:0030168 | platelet activation |
| GO:0110110 | positive regulation of animal organ morphogenesis |
| GO:0033555 | multicellular organismal response to stress |
| GO:0046887 | positive regulation of hormone secretion |
| GO:0003014 | renal system process |
| GO:0005796 | Golgi lumen |
| GO:0015696 | ammonium transport |
| GO:0042562 | hormone binding |
| GO:0031960 | response to corticosteroid |
| GO:0032526 | response to retinoic acid |
| GO:0051482 | positive regulation of cytosolic calcium ion concentration involved in phospholipase C-activating G protein-coupled signaling pathway |
| GO:0015026 | coreceptor activity |
| GO:0051781 | positive regulation of cell division |
| GO:0034767 | positive regulation of ion transmembrane transport |
| GO:0043271 | negative regulation of ion transport |
| GO:0022612 | gland morphogenesis |
| GO:0010469 | regulation of signaling receptor activity |
| GO:0032835 | glomerulus development |
| GO:0005003 | ephrin receptor activity |
| GO:0097755 | positive regulation of blood vessel diameter |
| GO:0005178 | integrin binding |
| GO:0045471 | response to ethanol |
| GO:1901343 | negative regulation of vasculature development |
| GO:0030879 | mammary gland development |
| GO:0051899 | membrane depolarization |
| GO:0035176 | social behavior |
| GO:0060076 | excitatory synapse |
| GO:0045776 | negative regulation of blood pressure |
| GO:0010543 | regulation of platelet activation |
| GO:0001540 | amyloid-beta binding |
| GO:0044070 | regulation of anion transport |
| GO:0060041 | retina development in camera-type eye |
| GO:0005245 | voltage-gated calcium channel activity |
| GO:0008361 | regulation of cell size |
| GO:0046888 | negative regulation of hormone secretion |
| GO:0004222 | metalloendopeptidase activity |
| GO:0062013 | positive regulation of small molecule metabolic process |
| GO:0004435 | phosphatidylinositol phospholipase C activity |
| GO:0031279 | regulation of cyclase activity |
| GO:0003044 | regulation of systemic arterial blood pressure mediated by a chemical signal |
| GO:0070405 | ammonium ion binding |
| GO:0043266 | regulation of potassium ion transport |
| GO:0048639 | positive regulation of developmental growth |
| GO:0051590 | positive regulation of neurotransmitter transport |
| GO:0032589 | neuron projection membrane |
| GO:0000186 | activation of MAPKK activity |
| GO:0015293 | symporter activity |
| GO:0014821 | phasic smooth muscle contraction |
| GO:0050805 | negative regulation of synaptic transmission |
| GO:0032355 | response to estradiol |
| GO:0090183 | regulation of kidney development |
| GO:1990782 | protein tyrosine kinase binding |
| GO:0045907 | positive regulation of vasoconstriction |
| GO:0046660 | female sex differentiation |
| GO:0055123 | digestive system development |
| GO:0001965 | G-protein alpha-subunit binding |
| GO:0099173 | postsynapse organization |
| GO:0002673 | regulation of acute inflammatory response |
| GO:0034776 | response to histamine |
| GO:0048708 | astrocyte differentiation |
| GO:0050728 | negative regulation of inflammatory response |
| GO:0019198 | transmembrane receptor protein phosphatase activity |
| GO:0007229 | integrin-mediated signaling pathway |
| GO:0070509 | calcium ion import |
| GO:0002792 | negative regulation of peptide secretion |
| GO:0090193 | positive regulation of glomerulus development |
| GO:0004725 | protein tyrosine phosphatase activity |
| GO:0051928 | positive regulation of calcium ion transport |
| GO:0060048 | cardiac muscle contraction |
| GO:0016048 | detection of temperature stimulus |
| GO:0050954 | sensory perception of mechanical stimulus |
| GO:0071248 | cellular response to metal ion |
| GO:0009582 | detection of abiotic stimulus |
| GO:0060037 | pharyngeal system development |
| GO:0002027 | regulation of heart rate |
| GO:0035264 | multicellular organism growth |
| GO:0007158 | neuron cell-cell adhesion |
| GO:0032370 | positive regulation of lipid transport |
| GO:0006970 | response to osmotic stress |
| GO:0048640 | negative regulation of developmental growth |
| GO:0001656 | metanephros development |
| GO:0050766 | positive regulation of phagocytosis |
| GO:0035108 | limb morphogenesis |
| GO:0098685 | Schaffer collateral - CA1 synapse |
| GO:0005518 | collagen binding |
| GO:0048813 | dendrite morphogenesis |
| GO:0007589 | body fluid secretion |
| GO:0055078 | sodium ion homeostasis |
| GO:0003071 | renal system process involved in regulation of systemic arterial blood pressure |
| GO:0015812 | gamma-aminobutyric acid transport |
| GO:0097306 | cellular response to alcohol |
| GO:0071236 | cellular response to antibiotic |
| GO:0008038 | neuron recognition |
| GO:0060349 | bone morphogenesis |
| GO:0001764 | neuron migration |
| GO:0045777 | positive regulation of blood pressure |
| GO:0035270 | endocrine system development |
| GO:0004715 | non-membrane spanning protein tyrosine kinase activity |
| GO:0003158 | endothelium development |
| GO:0010463 | mesenchymal cell proliferation |
| GO:0001696 | gastric acid secretion |
| GO:0030307 | positive regulation of cell growth |
| GO:0014902 | myotube differentiation |
| GO:0043279 | response to alkaloid |
| GO:0007190 | activation of adenylate cyclase activity |
| GO:0005901 | caveola |
| GO:0001569 | branching involved in blood vessel morphogenesis |
| GO:0051969 | regulation of transmission of nerve impulse |
| GO:0048010 | vascular endothelial growth factor receptor signaling pathway |
| GO:0042923 | neuropeptide binding |
| GO:0051965 | positive regulation of synapse assembly |
| GO:0035051 | cardiocyte differentiation |
| GO:0140253 | cell-cell fusion |
| GO:0033273 | response to vitamin |
| GO:1905330 | regulation of morphogenesis of an epithelium |
| GO:1904645 | response to amyloid-beta |
| GO:0090102 | cochlea development |
| GO:0050680 | negative regulation of epithelial cell proliferation |
| GO:0061614 | pri-miRNA transcription by RNA polymerase II |
| GO:0001709 | cell fate determination |
| GO:0031290 | retinal ganglion cell axon guidance |
| GO:0060080 | inhibitory postsynaptic potential |
| GO:0005024 | transforming growth factor beta-activated receptor activity |
| GO:0017146 | NMDA selective glutamate receptor complex |
| GO:0007588 | excretion |
| GO:0007223 | Wnt signaling pathway, calcium modulating pathway |
| GO:0030856 | regulation of epithelial cell differentiation |
| GO:0099604 | ligand-gated calcium channel activity |
| GO:0072089 | stem cell proliferation |
| GO:0007157 | heterophilic cell-cell adhesion via plasma membrane cell adhesion molecules |
| GO:0051967 | negative regulation of synaptic transmission, glutamatergic |
| GO:0060412 | ventricular septum morphogenesis |
| GO:0007213 | G protein-coupled acetylcholine receptor signaling pathway |
| GO:0016331 | morphogenesis of embryonic epithelium |
| GO:0030431 | sleep |
| GO:0010634 | positive regulation of epithelial cell migration |
| GO:2000249 | regulation of actin cytoskeleton reorganization |
| GO:0042632 | cholesterol homeostasis |
| GO:0033135 | regulation of peptidyl-serine phosphorylation |
| GO:0018958 | phenol-containing compound metabolic process |
| GO:0007492 | endoderm development |
| GO:0055075 | potassium ion homeostasis |
| GO:0140115 | export across plasma membrane |
| GO:0014013 | regulation of gliogenesis |
| GO:0009898 | cytoplasmic side of plasma membrane |
| GO:0060688 | regulation of morphogenesis of a branching structure |
| GO:0042383 | sarcolemma |
| GO:0071855 | neuropeptide receptor binding |
| GO:0098900 | regulation of action potential |
| GO:0003177 | pulmonary valve development |
| GO:0010745 | negative regulation of macrophage derived foam cell differentiation |
| GO:0045598 | regulation of fat cell differentiation |
| GO:0007219 | Notch signaling pathway |
| GO:0031092 | platelet alpha granule membrane |
| GO:0043198 | dendritic shaft |
| GO:0035591 | signaling adaptor activity |
| GO:0042752 | regulation of circadian rhythm |
| GO:0003148 | outflow tract septum morphogenesis |
| GO:0001963 | synaptic transmission, dopaminergic |
| GO:0045807 | positive regulation of endocytosis |
| GO:0010811 | positive regulation of cell-substrate adhesion |
| GO:0035924 | cellular response to vascular endothelial growth factor stimulus |
| GO:0007263 | nitric oxide mediated signal transduction |
| GO:0030136 | clathrin-coated vesicle |
| GO:0043949 | regulation of cAMP-mediated signaling |
| GO:0060976 | coronary vasculature development |
| GO:0007191 | adenylate cyclase-activating dopamine receptor signaling pathway |
| GO:0071679 | commissural neuron axon guidance |
| GO:0005161 | platelet-derived growth factor receptor binding |
| GO:0072224 | metanephric glomerulus development |
| GO:2000846 | regulation of corticosteroid hormone secretion |
| GO:0050905 | neuromuscular process |
| GO:0007422 | peripheral nervous system development |
| GO:0001779 | natural killer cell differentiation |
| GO:0003085 | negative regulation of systemic arterial blood pressure |
| GO:0071312 | cellular response to alkaloid |
| GO:0017124 | SH3 domain binding |
| GO:0007628 | adult walking behavior |
| GO:0005201 | extracellular matrix structural constituent |
| GO:0098815 | modulation of excitatory postsynaptic potential |
| GO:0072567 | chemokine (C-X-C motif) ligand 2 production |
| GO:0031100 | animal organ regeneration |
| GO:0045747 | positive regulation of Notch signaling pathway |
| GO:0002028 | regulation of sodium ion transport |
| GO:0072017 | distal tubule development |
| GO:0031128 | developmental induction |
| GO:0072202 | cell differentiation involved in metanephros development |
| GO:0005496 | steroid binding |
| GO:0060996 | dendritic spine development |
| GO:0048008 | platelet-derived growth factor receptor signaling pathway |
| GO:0007156 | homophilic cell adhesion via plasma membrane adhesion molecules |
| GO:0002026 | regulation of the force of heart contraction |
| GO:0035815 | positive regulation of renal sodium excretion |
| GO:0050892 | intestinal absorption |
| GO:0002065 | columnar/cuboidal epithelial cell differentiation |
| GO:0010718 | positive regulation of epithelial to mesenchymal transition |
| GO:0035137 | hindlimb morphogenesis |
| GO:0043267 | negative regulation of potassium ion transport |
| GO:0044304 | main axon |
| GO:0060158 | phospholipase C-activating dopamine receptor signaling pathway |
| GO:1903779 | regulation of cardiac conduction |
| GO:0021675 | nerve development |
| GO:0032060 | bleb assembly |
| GO:0072205 | metanephric collecting duct development |
| GO:0044325 | ion channel binding |
| GO:1901888 | regulation of cell junction assembly |
| GO:0002076 | osteoblast development |
| GO:0048148 | behavioral response to cocaine |
| GO:0043277 | apoptotic cell clearance |
| GO:0038127 | ERBB signaling pathway |
| GO:0005112 | Notch binding |
| GO:0006883 | cellular sodium ion homeostasis |
| GO:0048286 | lung alveolus development |
| GO:0061577 | calcium ion transmembrane transport via high voltage-gated calcium channel |
| GO:0021536 | diencephalon development |
| GO:1903428 | positive regulation of reactive oxygen species biosynthetic process |
| GO:0038003 | opioid receptor signaling pathway |
| GO:0005604 | basement membrane |
| GO:0002040 | sprouting angiogenesis |
| GO:0005184 | neuropeptide hormone activity |
| GO:0010996 | response to auditory stimulus |
| GO:0048146 | positive regulation of fibroblast proliferation |
| GO:0010766 | negative regulation of sodium ion transport |
| GO:0090075 | relaxation of muscle |
| GO:0050919 | negative chemotaxis |
| GO:0031594 | neuromuscular junction |
| GO:1900120 | regulation of receptor binding |
| GO:0034446 | substrate adhesion-dependent cell spreading |
| GO:0071402 | cellular response to lipoprotein particle stimulus |
| GO:0007179 | transforming growth factor beta receptor signaling pathway |
| GO:1904321 | response to forskolin |
| GO:0140058 | neuron projection arborization |
| GO:0001889 | liver development |
| GO:0046661 | male sex differentiation |
| GO:0010470 | regulation of gastrulation |
| GO:0005154 | epidermal growth factor receptor binding |
| GO:0097110 | scaffold protein binding |
| GO:0043534 | blood vessel endothelial cell migration |
| GO:0035023 | regulation of Rho protein signal transduction |
| GO:0001784 | phosphotyrosine residue binding |
| GO:0030513 | positive regulation of BMP signaling pathway |
| GO:0040018 | positive regulation of multicellular organism growth |
| GO:0032488 | Cdc42 protein signal transduction |
| GO:0032793 | positive regulation of CREB transcription factor activity |
| GO:0050966 | detection of mechanical stimulus involved in sensory perception of pain |
| GO:0051481 | negative regulation of cytosolic calcium ion concentration |
| GO:0043114 | regulation of vascular permeability |
| GO:0110111 | negative regulation of animal organ morphogenesis |
| GO:1900006 | positive regulation of dendrite development |
| GO:0003084 | positive regulation of systemic arterial blood pressure |
| GO:0010623 | programmed cell death involved in cell development |
| GO:0030427 | site of polarized growth |
| GO:0042908 | xenobiotic transport |
| GO:0043954 | cellular component maintenance |
| GO:0044331 | cell-cell adhesion mediated by cadherin |
| GO:0045742 | positive regulation of epidermal growth factor receptor signaling pathway |
| GO:0050853 | B cell receptor signaling pathway |
| GO:0020037 | heme binding |
| GO:0045986 | negative regulation of smooth muscle contraction |
| GO:0035633 | maintenance of permeability of blood-brain barrier |
| GO:0048407 | platelet-derived growth factor binding |
| GO:1990138 | neuron projection extension |

* Marked in green: morphogenesis and development related gene sets, blue: gene sets related to neural processes, green/blue: gene sets related to both

**Supplementary Table S6:** Degree, **c**loseness and betweenness centrality of the top ranking genes of morphogenesis and development in a SARS-CoV-2 specific protein-protein interaction network

| Gene symbol | Degree in a SARS-CoV-2 specific PPI^1^ | Closeness centrality in a SARS-CoV-2 specific PPI^1-3^ | Betweenness centrality in a SARS-CoV-2 specific PPI^1-3^ | Quantile gene expression^4^ |
| --- | --- | --- | --- | --- |
| APP | 110 | 0.439 | 0.174 | 91 |
| TP53 | 66 | 0.426 | 0.079 | 82 |
| AKT1 | 48 | 0.416 | 0.041 | 93 |
| ERBB2 | 51 | 0.405 | 0.048 | 79 |
| EP300 | 60 | 0.366 | 0.048 | 74 |
| SRC | 42 | 0.406 | 0.035 | 97 |
| SMAD3 | 44 | 0.389 | 0.039 | 97 |
| JUN | 23 | 0.399 | 0.018 | 76 |
| NOTCH1 | 23 | 0.380 | 0.018 | 63 |
| NCOR2 | 27 | 0.371 | 0.017 | 82 |
| CTNNB1 | 20 | 0.394 | 0.014 | 98 |
| RACK1 | 18 | 0.386 | 0.019 | 99 |
| STAT3 | 29 | 0.356 | 0.022 | 94 |
| PTPN11 | 24 | 0.360 | 0.010 | 95 |
| AR | 27 | 0.352 | 0.013 | 93 |
| MET | 21 | 0.390 | 0.005 | 98 |
| HDAC1 | 29 | 0.342 | 0.014 | 58 |
| NR3C1 | 27 | 0.348 | 0.012 | 99 |
| PPARG | 23 | 0.348 | 0.012 | 74 |
| CAV1 | 13 | 0.378 | 0.006 | 97 |

1) Based on a protein interaction network of developmental genes in which protein interactions of SARS-CoV-2 non-structural proteins with host proteins have been added

2) Determined using the Python package Networkx

3) Decimal places were rounded

4) Based on the expression levels of the listed gene across several time points of SARS-CoV-2 infected A549 cells (details, see Methods)

**Supplementary Table S7:** IC_50_ determination by non-linear regression for infection in A549-AT

| *Classification* | *Virus* | *IC_50_ PRI-724 [µM]* | *IC_50_ Remdesivir [µM]* |
| --- | --- | --- | --- |
| Ancestral | B (FFM5) | 1.869 | 0.6051 |
|  | B.1 (FFM7, D614G) | 2.881 | 0.569 |
|  | SARS-CoV-1 (FFM1) | 1.435 | 0.2305 |
| VOI | B.1.429 (Epsilon) | 2.345 | 0.4271 |
|  | P.2 (Zeta) | 1.496 | 0.2487 |
|  | B.1.617.1 (Kappa) | 1.527 | 0.1976 |
| Deescalated VOC | B.1.1.7 (Alpha) | 1.183 | 0.1947 |
|  | B.1.351 (Beta) | 1.471 | 0.2211 |
|  | B.1.617.2 (Delta) | 1.369 | 0.227 |
| VOC | B.1.1.529 BA.1 (Omicron) | 0.8123 | 0.19 |
|  | B.1.1.529 BA.2 (Omicron) | 0.7522 | 0.1979 |
|  | B.1.1.529 BA.5 (Omicron) | 0.7563 | 0.1910 |
| Total | *Sarbecovirus* | **1.491**  (95%CI 1.087-1.896) | **0.2916**  (95%CI 0.1948-0.3885) |
| Coefficient of variation (CV) | *Sarbecovirus* | **42.72%** | **52.28%** |

**Supplementary Table S8:** IC_50_ determination by non-linear regression for infection in Calu-3

| *Classification* | *Virus* | *IC_50_ PRI-724 [µM]* | *IC_50_ Remdesivir [µM]* |
| --- | --- | --- | --- |
| Ancestral | B.1 (FFM7, D614G) | 7.824 | 0.403 |
| VOI | P.2 (Zeta) | 7.081 | 0.379 |
| Deescalated VOC | B.1.1.7 (Alpha) | 9.400 | 0.321 |
|  | B.1.351 (Beta) | 8.264 | 0.629 |
|  | B.1.617.2 (Delta) | 9.671 | 0.749 |
| Total | SARS-CoV-2 | **8.448**  (95%CI 7.431-9.465) | **0.496**  (95%CI 0.269-0.724) |
| Coefficient of variation (CV) | SARS-CoV-2 | **11.47%** | **36.94%** |

**Supplementary Table S9:** Two-way ANOVA for comparison of vRNA levels upon treatments

SARS-CoV-2, A549-AT

| *comp. DMSO to* | hours post infection (hpi) | | | | | | |
| --- | --- | --- | --- | --- | --- | --- | --- |
|  | 2 | 4 | 6 | 8 | 10 | 12 | 24 |
| Remdesivir | 0,0543  (2.8x) | 0,2136  (1.6x) | 0,0288  (14.5x) | 0,1233  (43.4x) | 0,0388  (67.7x) | 0,0270  (98.7x) | 0,0173  (270x) |
| 10 µM PRI | 0,0709  (2.3x) | 0,0392  (240.6x) | 0,0271  (370.2x) | 0,1212  (76.1x) | 0,0388  (59.2x) | 0,0271  (76.5x) | 0,0175  (95.8x) |
| 3 µM PRI | 0,0628  (2.4x) | 0,0457  (11.9x) | 0,0283  (37.8x) | 0,1308  (17.2x) | 0,0488  (6.9x) | 0,0314  (9.1x) | 0,0193  (13.3x) |
| 1 µM PRI | 0,0690  (2.0x) | 0,0563  (4.9x) | 0,0288  (20.0x) | 0,1450  (8.3x) | 0,0496  (3.9x) | 0,0368  (5.6x) | 0,0100  (7.2x) |

SARS-CoV-1, A549-AT

| *comp. DMSO to* | hours post infection (hpi) | | | | | | |
| --- | --- | --- | --- | --- | --- | --- | --- |
|  | 2 | 4 | 6 | 8 | 10 | 12 | 24 |
| Remdesivir | 0,2245  (1.6x) | 0,2140  (2.6x) | 0,1388  (41.7x) | 0,0387  (50.9x) | 0,0223  (26.1x) | 0,0078  (38.0x) | 0,0164  (3142x) |
| 10 µM PRI | 0,1002  (2.6x) | 0,1030  (61.4x) | 0,1348  (166.7x) | 0,0375  (594.6x) | 0,0209  (242.6x) | 0,0079  (94.0x) | 0,0164  (1287.6x) |
| 3 µM PRI | 0,0720  (4.0x) | 0,1058  (31.2x) | 0,1345  (213.2x) | 0,0386  (54.7x) | 0,0233  (16.9x) | 0,0083  (14.5x) | 0,0166  (156.6x) |
| 1 µM PRI | 0,8730  (0.9x) | 0,1115  (15.4x) | 0,1360  (86.9x) | 0,0421  (7.8x) | 0,0259  (7.5x) | 0,0089  (7.9x) | 0,0178  (22.4x) |

SARS-CoV-2, Calu-3

| *comp. DMSO to* | hours post infection (hpi) | | | | | | |  |
| --- | --- | --- | --- | --- | --- | --- | --- | --- |
|  | 4 | 6 | 8 | 10 | 12 | 24 | 36 | 48 |
| Remdesivir | 0,0561  (4.3x) | 0,0651  (9.1x) | 0,0155  (10.6x) | 0,0753  (5.7x) | 0,1831  (4.5x) | 0,0941  (114.6x) | 0,0148  (248.9x) | 0,0093  (244.4x) |
| 20 µM PRI | 0,0548  (4.0x) | 0,0739  (5.5x) | 0,0141  (2.9x) | 0,1821  (1.9x) | 0,2387  (2.8x) | 0,0980  (31.5x) | 0,0150  (104.6x) | 0,0097  (36.8x) |
| 15 µM PRI | 0,0446  (8.4x) | 0,0623  (12.5x) | 0,0230  (4.3x) | 0,2337  (1.7x) | 0,5171  (1.4x) | 0,0979  (31.9x) | 0,0151  (69.1x) | 0,0098  (12.9x) |
| 10 µM PRI | 0,0452  (7.6x) | 0,0612  (10.5x) | 0,0182  (3.5x) | 0,3589  (1.4x) | 0,5082  (1.5x) | 0,1040  (15.4x) | 0,0169  (9.7x) | 0,0118  (2.8x) |

**In each cell of the table, individual p values are listed for the comparison between DMSO and a respective treatment at each timepoint. Significant results are marked in green. Underneath in brackets, rounded factors between RNA levels of control and respective treatments are listed.*

**Supplementary Table S10:** Sequences of primers used in qRT-PCR experiments

|  | Sequence |  | 3´ modification | Target | References |
| --- | --- | --- | --- | --- | --- |
| P1 | AACCAACCTCGATCTCTTGT | fwd | none | sg-N (SARS-CoV-1) |  |
| P2 | TCCACCAAATGTAATGCGG | rev | none | sg-N (SARS-CoV-1) |  |
| P3 | TGGACCCCAATCAAACCAACGT | - | BHQ-1 | sg-N (SARS-CoV-1) |  |
| P4 | AAGAAATTCAACTCCTGGCA | fwd | none | N (SARS-CoV-1) |  |
| P5 | GCTGGTTCAATCTGTCTAGC | rev | none | N (SARS-CoV-1) |  |
| P6 | AGCGGAGGTGGTGAAACTGC | - | BHQ-3 | N (SARS-CoV-1) |  |
| P7 | AACCAACCAACTTTCGATCTC | fwd | none | sg-N (SARS-CoV-2) | (1) |
| P8 | AGTTGAATCTGAGGGTCCAC | rev | none | sg-N (SARS-CoV-2) | (1) |
| P9 | TGGACCCCAAAATCAGCGAAATGC | - | BHQ-1 | sg-N (SARS-CoV-2) | (1) |
| P10 | GGGACCAGGAACTAATCAGA | fwd | none | N (SARS-CoV-2) | (1) |
| P11 | TGTGACTTCCATGCCAATG | rev | none | N (SARS-CoV-2) | (1) |
| P12 | GCGTTCTTCGGAATGTCGCG | - | BHQ-2 | N (SARS-CoV-2) | (1) |
| P13 | AGATTTGGACCTGCGAGCG | fwd | none | RNase P Exon 1 | (2) |
| P14 | GAGCGGCTGTCTCCACAAGT | rev | none | RNase P | (2) |
| P15 | TTCTGACCTGAAGGCTCTGCGCG | - | BHQ-3 | RNase P | (2) |
| P16 | CTCCTCCGAGTCAACAGATTCA | fwd | none | CDC25A  PrimerBank ID 42490757c2 | (3) |
| P17 | CAACAGCTTCTGAGGTAGGGA | rev | none | CDC25A | (3) |
| P18 | AGGACCACCGCATCTCTACAT | fwd | none | BIRC5  PrimerBank ID 59859879c1 | (3) |
| P19 | AAGTCTGGCTCGTTCTCAGTG | rev | none | BIRC5 | (3) |

**Supplementary Table S11:** List of primary and secondary antibodies

| # | Target | Dilution | Purchase information |
| --- | --- | --- | --- |
| PA1 | SARS-CoV/SARS-CoV-2 N | 1:1000 | Sino Biological (#40143-MM05) |
| PA2 | Influenza A Nucleoprotein | 1:1000 | Merck (#MAB8251) |
| PA3 | c-Jun | 1:1000 | Cell signalling (#9165) |
| PA4 | Cyclin D1 | 1:1000 | Cell signalling (#2978) |
| PA5 | β-catenin | 1:1000 | Cell signalling (#8480) |
| PA6 | β-actin | 1:1000 | Cell signalling (#4967S) |
| SA1 | anti-mouse IgG (A488) | 1:1000 | Invitrogen (#WF319853) |
| SA2 | anti-mouse IgG (A647) | 1:1000 | Invitrogen (#WE322197) |
| SA3 | IRDye 680RD goat anti-mouse IgG | 1:25,000 | LI-COR (#926-68070) |
| SA4 | IRDye 800CW goat anti-rabbit IgG | 1:25,000 | LI-COR (#926-32211) |

**Supplemental Videos 1-6**

Video S1: 3 µM PRI-724, SARS-CoV-2 (B.1.617.2 isolate)

Video S2: 1 µM PRI-724, SARS-CoV-2 (B.1.617.2 isolate)

Video S3: DMSO, SARS-CoV-2 (B.1.617.2 isolate)

Video S4: 3 µM PRI-724, SARS-CoV-1 (Frankfurt-1 isolate)

Video S5: 1 µM PRI-724, SARS-CoV-1 (Frankfurt-1 isolate)

Video S6: DMSO, SARS-CoV-1 (Frankfurt-1 isolate)

**References**

1. Veleanu A, Kelch MA, Ye C, Flohr M, Wilhelm A, Widera M, et al. Molecular Analyses of Clinical Isolates and Recombinant SARS-CoV-2 Carrying B.1 and B.1.617.2 Spike Mutations Suggest a Potential Role of Non-Spike Mutations in Infection Kinetics. Viruses. 2022;14(9).

2. Molecular Assays to Diagnose COVID-19: Summary Table of Available Protocols [Internet]. 2020. Available from: Available online: <https://www.who>.int/docs/default-source/coronaviruse/whoinhouseassays.pdf?sfvrsn=de3a76aa_2.

3. Spandidos A, Wang X, Wang H, Dragnev S, Thurber T, Seed B. A comprehensive collection of experimentally validated primers for Polymerase Chain Reaction quantitation of murine transcript abundance. BMC Genomics. 2008;9:633.
